# (1→3)- β-D-Glucan Levels Correlate With Neurocognitive Functioning in HIV-Infected Persons on Suppressive Antiretroviral Therapy: A Cohort Study: Erratum

**DOI:** 10.1097/01.md.0000484041.05495.82

**Published:** 2016-05-20

In the article “(1→3)- β-D-Glucan Levels Correlate With Neurocognitive Functioning in HIV-Infected Persons on Suppressive Antiretroviral Therapy: A Cohort Study”,^1^ which appeared in Volume 95, Issue 11 of *Medicine*, the degrees of Drs. de Oliveira, Finkelman, and Marcotte were misreported as MDs. All three authors have PhDs. The article has since been corrected online.
